# *KRAS/BRAF* Analysis in Ovarian Low-Grade Serous Carcinoma Having Synchronous All Pathological Precursor Regions

**DOI:** 10.3390/ijms17050625

**Published:** 2016-04-26

**Authors:** Kohei Nakamura, Kentaro Nakayama, Tomoka Ishibashi, Noriyoshi Ishikawa, Masako Ishikawa, Hiroshi Katagiri, Toshiko Minamoto, Emi Sato, Kaori Sanuki, Hitomi Yamashita, Kouji Iida, Razia Sultana, Satoru Kyo

**Affiliations:** 1 Department of Obstetrics and Gynecology, Shimane University School of Medicine, Izumo 6938501, Japan; kohei320@med.shimane-u.ac.jp (K.N.); tomoka19850314@gmail.com (T.I.); pcbashi4@yahoo.co.jp (M.I.); hkata@med.shimane-u.ac.jp (H.K.); tokominmin@gmail.com (T.M.); prettynanaone@gmail.com (E.S.); kaorisanuki@gmail.com (K.S.); memedasudasu1103@gmail.com (H.Y.); iida@med.shimane-u.ac.jp (K.I.); raeedahmed@yahoo.com (R.S.); satoruky@med.shimane-u.ac.jp (S.K.); 2 Department of Organ Pathology, Shimane University School of Medicine, Izumo 6938501, Japan; kanatomo@med.shimane-u.ac.jp

**Keywords:** low-grade serous carcinoma, *KRAS*, *BRAF*, mutation

## Abstract

Ovarian low-grade serous carcinoma is thought to begin as a serous cystadenoma or adenofibroma that progresses in a slow stepwise fashion. Among the low-grade serous carcinomas, there is a high frequency of activating mutations in the *KRAS* or *BRAF* genes; however, it remains unclear as to how these mutations contribute to tumor progression. This is the first report to track the histopathological progression of serous adenofibroma to low-grade serous carcinoma. Each stage was individually analyzed by pathological and molecular genetic methods to determine what differences occur between the distinct stages of progression.

## 1. Introduction

Serous ovarian carcinoma is the eighth most common cancer in women with approximately 140,000 deaths per year worldwide [1]. Recent studies divide ovarian carcinomas into two groups, designated Types I and II [2]. Type II tumors include the high-grade serous carcinomas, which are associated with TP53 mutations and a rapidly progressive clinical course. Type I tumors occur less frequently than Type II. This group includes low-grade serous carcinoma, low-grade endometrioid carcinoma, clear cell carcinoma, mucinous carcinoma and malignant Brenner tumor. Two-thirds of these Type I low-grade serous carcinoma cases are associated with Kirsten rat sarcoma viral oncogene homolog (*KRAS*), B-Raf proto-oncogene (*BRAF*) or erb-b2 receptor tyrosine kinase 2 (*ERRB2*) mutations. Type I tumors also display a characteristic slow tempo of tumor development and a low level of chromosomal instability.

Until recently, the pathway of development of low-grade serous carcinomas was not clear. Shih and Kurman [2,3] found that low-grade serous carcinoma (invasive focus of micropapillary serous carcinoma) arises in a slow stepwise fashion: a serous cystadenoma or adenofibroma progresses to an atypical proliferative (borderline) serous tumor (APST) to noninvasive micropapillary serous borderline tumor (noninvasive MPSC) and, then, to low-grade serous carcinoma [2,3]. *KRAS* and *BRAF* mutations are not found in the early serous cystadenoma stage [4], but they have been detected in the APSTs and adjacent cystadenoma epithelium in serous cystadenomas associated with small APSTs [5]. These findings suggest that acquisition of *KRAS* or *BRAF* mutations may relate to progression from cystadenoma or adenofibroma to APST.

The pathogenesis, clinicopathologic and molecular analysis of different stages are described in some studies [4,5], but no study has included a detailed analysis of all stages leading to low-grade serous carcinoma, since it is extremely rare to find one case displaying all stages. This is the first report of a single case of low-grade serous carcinoma exhibiting serous adenofibroma, APST, noninvasive MPSC and low-grade serous carcinoma. Each distinct region was analyzed to determine differences in pathology and molecular genetics.

## 2. Results

In this tumor, we found various histopathological stages of progression to low-grade serous carcinoma, including serous adenofibroma, APST, noninvasive MPSC and low-grade serous carcinoma. We also identified a small amount of noninvasive peritoneal epithelial implant and para-aortic lymph node lesion associated with atypical proliferative serous tumor.

### 2.1. Pathological Findings

**Figure 1:**
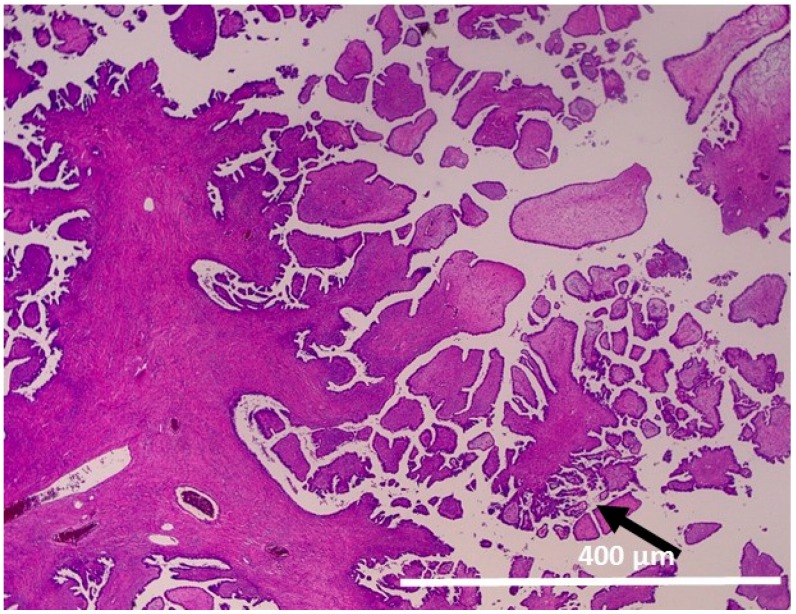
Serous adenofibroma with papillary proliferation without atypia. Papillary growth with a core of broad fibrous stroma (100×). Papillary structures are increasing in some areas (arrow).

Serous adenofibroma with papillary proliferation without atypia:

The tumor showed papillary growth with a core of broad fibrous stroma, and a micropapillary structure of the papillary region was also seen (Figure 1).

#### 2.1.1. Atypical Proliferative Serous Tumor (APST)

**Figure 2:**
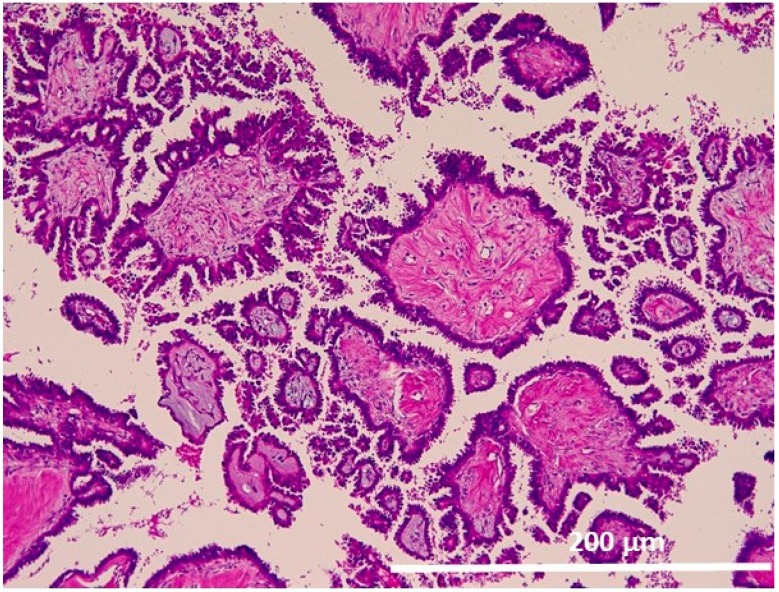
Atypical proliferative serous tumor (APST). Fibrous papillae with numerous smaller daughter papillae projected into cystic spaces (200×).

The tumor cells displayed extensive epithelial stratification, tufting and detachment of individual cells and cell clusters in addition to hierarchical branching with numerous smaller daughter papillae projected into cystic spaces (Figure 2). 

#### 2.1.2. Noninvasive Noninvasive Micropapillary Serous Borderline Tumor (MPSC)

**Figure 3:**
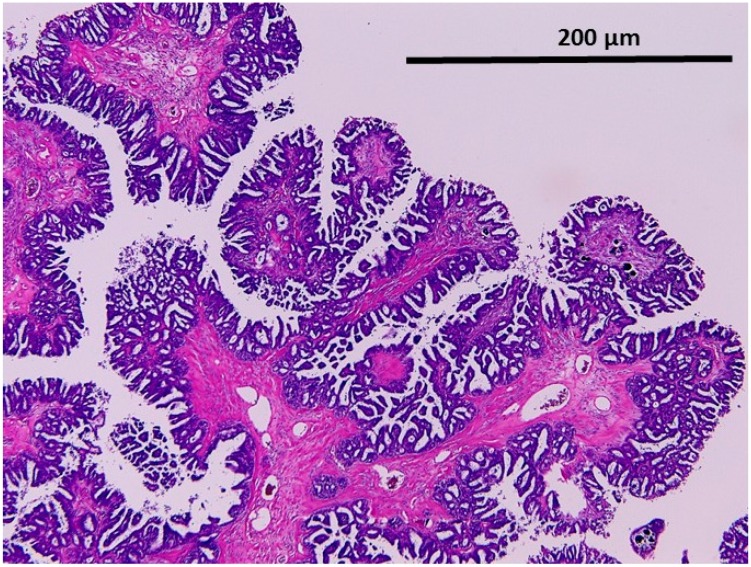
Noninvasive micropapillary serous carcinoma (noninvasive MPSC). Epithelial proliferation and complexity with micropapillary pattern (200×).

The tumor cells displayed high degrees of epithelial proliferation and complexity with a micropapillary pattern. A myriad of delicate micropapillae with thin or no fibrovascular cores radiate from more broad fibrovascular cores (Figure 3). 

#### 2.1.3. Low-Grade Serous Carcinoma (Invasive Focus of MPSC)

**Figure 4:**
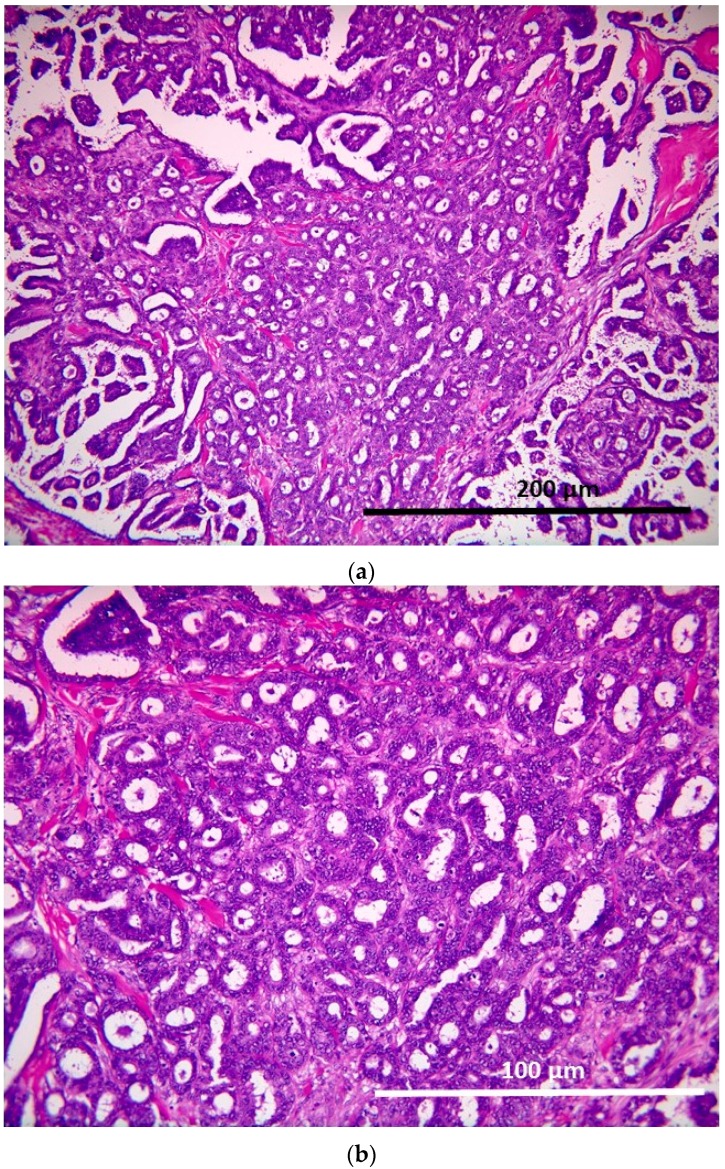
(**a**) Low-grade serous carcinoma. The haphazard infiltrative growth with micropapillae (200×); (**b**) Low-grade serous carcinoma. The haphazard infiltrative growth with micropapillae (400×).

The tumor cells displayed haphazard infiltrative growth composed of small glands with a background of the micropapillary component (Figure 4a,b). 

### 2.2. Genetic Analysis of Distinct Tumor Regions

Polymerase chain reaction (PCR) amplification and sequence analysis were successful in the regions of serous adenofibroma, APST, noninvasive MPSC and low-grade serous carcinoma. The implants and lymph node were so small that PCR amplification could not be performed. All regions that could be analyzed were wild-type at the mutation hot spots for both *KRAS* and *BRAF* genes (Table 1).

## 3. Discussion

Low-grade serous carcinomas are genetically stable and characterized by their low number of genetic mutations, which may account for the slow stepwise development of these tumors. In some studies, low-grade serous carcinoma is considered to arise from a serous cystadenoma or adenofibroma, which progresses to APST, to noninvasive MPSC and, then, to low-grade serous carcinoma [2,3]. Malpica *et al.* [6] found that low-grade serous carcinoma is associated with noninvasive MPSC. Smith, Sehdev *et al.* [7] showed that true early invasion in APST or noninvasive MPSC resembles low-grade serous carcinoma. Furthermore, noninvasive MPSCs have a higher frequency of invasive implants compared to APSTs, and these implants are histologically identical to low-grade serous carcinomas [8,9]. They are associated with *KRAS*, *BRAF* or *ERRB2* mutations in two-thirds of cases [2,9,10,11]. Furthermore, *ERBB2* mutations occur less frequently compared to *KRAS* and *BRAF* mutations, and *KRAS* and *BRAF* mutations are mutually exclusive.

Our report has demonstrated for the first time that all regions of adenofibroma, APST, noninvasive MPSC and low-grade serous carcinoma could be detected in one case. The coexistence of serous adenofibroma, APST, noninvasive MPSC and low-grade serous carcinoma strongly suggests that the adenofibromas are putative precursors of low-grade serous carcinomas. Sequence analysis revealed no genetic mutations of *BRAF* or *KRAS* hotspots, which does not support the hypothesis that mutations of *KRAS* and *BRAF* occur at the development of adenofibromas to APSTs. This suggests that, in this case, epigenetic changes may be associated with the development of the cancer. Furthermore, the frequency of *KRAS* and *BRAF* mutations in Japanese individuals with low-grade serous carcinomas is not known. Interestingly, in a previous study, *KRAS* (but not *BRAF*) mutation in ovarian serous borderline tumor is associated with recurrent low-grade serous carcinoma [12]. This leaves open the possibility that low-grade serous carcinomas develop by different means depending on the genetic make-up of the individual. We are currently investigating the frequency of these genetic mutations in Japanese.

Recent studies divide ovarian carcinomas into two groups, designated Types I and II, and Type II tumors are associated with TP53 mutations. Meanwhile, several studies revealed that TP53 mutations were not only found in Type II high grade serous, but also in Type I mucinous borderline tumor or carcinomas (>50% with p53 mutations) [13]. From these facts, it is difficult to classify ovarian carcinoma by only the detected genetic mutations.

## 4. Materials and Methods

### 4.1. Tissue Sample

Formalin-fixed, paraffin-embedded (FFPE) tissue samples of a low-grade serous carcinoma were used in this study. These specimens included adenofibroma, APST, noninvasive MPSC, low-grade serous carcinoma, a noninvasive peritoneal implant and metastasis to lymph node.

Samples were obtained from the Department of Obstetrics and Gynecology at the Shimane University Hospital (Izumo, Japan). The patient of this case was a 58-year-old gravida 2, para 2 woman with a chief complaint of abdominal distension. She underwent total abdominal hysterectomy with bilateral salpingo-oophorectomy and omentectomy. The resected specimens were reviewed by a pathologist, and the mass was finally diagnosed as stage IIIc, low-grade serous carcinoma. The specimens included serous adenofibroma, APST, noninvasive MPSC, low-grade serous carcinoma, noninvasive peritoneal implant and para-aortic lymph node associated with APST. Diagnosis was based on the conventional morphological examination of sections stained with hematoxylin and eosin (H & E). Acquisition of tissue specimens was approved by an institutional review board (No: 2009-0435, 22.03.2009, Shimane University Hospital).

### 4.2. Laser Capture Microdissection and DNA Extraction

All regions of adenofibroma, APST, noninvasive MPSC and low-grade serous carcinoma contained sufficient tumor tissue to extract DNA and perform sequence analysis. The regions of the non-invasive peritoneal implant and lymph node were too small to extract DNA. Tissue sections were placed on membrane slides and counterstained with hematoxylin. Selected tumor tissues were laser microdissected and captured using the PALM laser capture microdissection microscope (Leica Microsistem, LMD 7000, (Tokyo, Japan)). Approximately 500–4000 cells were microdissected for each ovarian or extraovarian region. After 48 h of proteinase digestion, DNA was extracted from the microdissected samples using a QIAamp DNA Micro Kit (Qiagen, Valencia, CA, USA).

### 4.3. Sequence Analysis

Jones *et al.* [14] demonstrated that APSTs and low-grade serous carcinomas rarely contain somatic mutations, except in *KRAS* at exon 2, codons 12–13 and in *BRAF* at exon 15, codon 600. PCR amplification was performed on these regions using genomic DNA from laser-captured microdissected FFPE tissue with the following primers: for exon 15 of *BRAF*, forward 5’-TGCTTGCTCTGATAGGAAAATGA-3’, reverse 5’-CCACAAAATGGATCCAGACAAC-3’; for exons 2–3 of *KRAS*, forward 5’-TAAGGCCTGCTGAAAATGACTG-3’, reverse 5’-TGGTCCTGCACCAGTAATATGC-3’. Amplified PCR products were sequenced at Beckman Coulter (Danvers, MA, USA) and analyzed with the Mutation Surveyor DNA Variant Analysis Software (Tokyo, Japan). Sequence analysis was successful in all specimens, except for the implants and the lymph node.

## 5. Conclusions

In summary, our experience supports the hypothesis that low-grade serous carcinoma is the end product of a progression of pathologies beginning with serous cystadenoma or adenofibroma that evolves through APST and noninvasive MPSC. Furthermore, epigenetic changes may be an important event in the carcinogenesis of some low grade serous carcinoma without *KRAS* or *BRAF* mutation.

## Figures and Tables

**Table 1:** *KRAS* and *BRAF* sequence analysis from distinct tumor regions.

Gene	Serous Adenofibroma	APST	Noninvasive MPSC	Low-Grade Serous Carcinoma	Noninvasive Implant	Lymph Node Associated with APST
*KRAS*	WT	WT	WT	WT	NA	NA
*BRAF*	WT	WT	WT	WT	NA	NA

APST: atypical proliferative serous tumor; noninvasive MPSC: noninvasive micropapillary serous borderline tumor; WT: wild-type; NA: .not available.
